# Investigation of the Magnesium Content and Productivity of Wheat Genotypes Under Organic and Conventional Inorganic Fertilizer Application

**DOI:** 10.3390/life15040543

**Published:** 2025-03-26

**Authors:** Essam M. Abd-Elmoniem, Nasser S. Al-Ghumaiz, Mohamad I. Motawei, Soleman Al-Otayk, Mokded Rabhi

**Affiliations:** 1 Department of Environment & Natural Resources, College of Agriculture & Food, Qassim University, Buraydah 52571, Saudi Arabia; emoniem@qu.edu.sa; 2 Department of Plant Production, College of Agriculture & Food, Qassim University, Buraydah 52571, Saudi Arabia; satiek@qu.edu.sa (S.A.-O.); m.rabhi@qu.edu.sa (M.R.)

**Keywords:** wheat, plants, magnesium, crop genetic diversity, conventional fertilization, organic fertilization

## Abstract

This study investigates Mg concentrations and productivity in seven spring wheat genotypes (YR, Local, Sids 12, P3, P5, IC8, and IC17) by evaluating their nutritional content and their responses to organic and conventional fertilization methods. We employed a randomized complete block design (RCBD) with three replications and observed that conventional fertilization resulted in higher Mg levels than organic fertilization (2.12 vs. 1.54 g kg^−1^). The application of conventional fertilizers also resulted in a higher shoot dry weight compared with the application of organic fertilizers (4.6 vs. 1.88 g), with Sids 12 recording the highest shoot dry weight (4.79 g), followed by YR (3.39 g). Furthermore, conventional fertilization consistently yielded a higher grain output than that of organic fertilization across both seasons. P5 and IC17 had superior grain yield and Mg content in grains, respectively. Wheat yields were lower under organic fertilization than under conventional practices. Some genotypes, such as YR and IC17, experienced significant yield reductions under organic conditions, whereas others, such as P5, displayed resilience or even enhanced yields. The IC17 genotype demonstrated minimal variation in Mg content in grains between conventional and organic fertilization, highlighting genotype-specific responses to fertilization methods. Thus, selecting and cultivating the appropriate genotype can facilitate achieving nutritionally adequate wheat production under organic farming conditions in Saudi Arabia.

## 1. Introduction

Globally, wheat (*Triticum aestivum* L., 2n = 6× = 42, AABBDD) is one of the most vital cereal crops and serves as a major source of magnesium (Mg) in human and animal nutrition [1]. Sandy soils exhibit markedly low fertility due to a pronounced deficiency in essential nutrients, including magnesium [2]. Consequently, the supplementation of magnesium to agricultural soils has become imperative. A significant proportion of the soils in the Qassim region are classified as sandy [3]. The application of organic manures is gaining prominence as an alternative to synthetic fertilizers, which are associated with environmental and health concerns. Recent studies by Al-Ghumaiz et al. [4] demonstrated that the incorporation of organic fertilizers into sandy soils enhanced wheat yield and increased the concentrations of selenium and zinc compared to conventional fertilization practices. Furthermore, Kumar et al. [5] highlighted that organic manures derived from both plant and animal sources offer a balanced nutrient profile that improves soil structure, enhances water retention capacity, and stimulates microbial activity. These manures facilitate the gradual release of nutrients, thereby reducing the risk of nutrient leaching and ensuring prolonged nutrient availability for crops. Additionally, organic manures promote the proliferation of beneficial soil microorganisms, which play a critical role in nutrient cycling and disease suppression. They also enhance the soil’s cation exchange capacity (CEC), which is vital for the retention and exchange of nutrients between the soil and plants. Collectively, these attributes contribute to the sustainability and long-term fertility of agricultural systems.

Several physiological functions have been attributed to magnesium in biological systems [6]. In plants, Mg is involved in a multitude of metabolic processes and reactions, including protein synthesis; chlorophyll formation; photophosphorylation; photosynthetic carbon dioxide (CO_2_) fixation in C3, C4, and CAM plants; photo-assimilate management (phloem loading, partitioning, and utilization); as well as in alleviating photo-oxidation and reactive oxygen species accumulation in leaf tissues [7]. Furthermore, magnesium constitutes the central atom of chlorophyll, where it facilitates photon capture in pigment–protein complexes within photosystem I (PSI) and photosystem II (PSII). Beyond its role in light absorption within the chlorophyll tetrapyrrole ring, Mg is integral to the CO_2_ assimilation processes in the chloroplast as well as the photoassimilation transport from leaves to non-photosynthetic organs. Approximately 75% of leaf cell Mg is directly or indirectly associated with protein biosynthesis, primarily through its involvement in ribosomal structure and function. Additionally, it plays a critical role in photophosphorylation, photosynthetic CO_2_ fixation, metabolic processes, and the partitioning and utilization of photoassimilates [7,8,9,10]. It also acts as a cofactor and allosteric regulator for over 300 enzymes, including carboxylases, phosphatases, protein kinases, RNA polymerases, and ATPases, and it serves as a regulator of the cation–anion balance and acts as an osmotically active ion that helps maintain cell turgor alongside potassium [8,9].

According to Ceylan et al. [11], insufficient Mg supply in wheat does not affect vegetative biomass but significantly reduces grain yield by limiting carbohydrate availability. They also showed that applying foliar Mg sprays after anthesis can significantly alleviate yield losses caused by Mg deficiency. Tränkner and Jaghdani [12] further demonstrated that a magnesium tissue concentration of 1.5 mg g^−1^ dry matter (DM) did not adversely affect the photosynthetic capacity of wheat and sunflower leaves. Significant reductions in Mg concentrations in cereal grains have been documented over the past few decades, primarily attributed to yield dilution effects associated with the Green Revolution [13]. Optimal plant growth requires Mg concentrations of 1.5–3.5 g kg^−1^ in vegetative tissues, with soil solution Mg levels ranging from 125 μmol L^−1^ to 8.5 mmol L^−1^ [8,14]. Symptoms of leaf chlorosis were observed in Mg-deficient bean plants grown at high light intensity. The green portion of the leaves was partially shaded with filter paper. With an adequate Mg supply, high light did not cause any leaf chlorosis [15]. Mg deficiency is a common nutritional disorder in plants and a widespread problem affecting crop productivity and quality [16]. About 90–98% of the soil Mg is contained in the crystal lattice structure of minerals and not directly available to plants [17]. The only existing form of Mg for uptake is Mg^2+^, which has the lowest ionic radius and the biggest hydrated radius among different cations [18]. This unique chemical property creates a weak bond between Mg^2+^ and negatively charged soil colloids as well as root cell borders, favoring the deficiency of interchangeable Mg from the soil.

Many countries aim to cultivate Mg-rich wheat varieties [19]. However, Saudi Arabia faces challenges in this owing to Mg-deficient soils, a problem that is exacerbated by the extensive use of potassium fertilizers. In calcareous soils, the presence of calcium and bicarbonates significantly hinders Mg uptake, leading to depletion [20], whereas in alkaline soils, the availability of Mg is limited due to the formation of magnesium carbonate and gypsum [21]. A substantial decline in cereal grain Mg concentrations over recent decades has been associated with the Green Revolution, with an average decrease of 19.6% in wheat Mg content from before to after 1968 [22]. Although Mg has historically been overlooked in fertilization strategies, recent expert analyses have highlighted its importance, equating it to that of nitrogen, potassium, and phosphorus.

Previous studies conducted in Saudi Arabia have focused on exploring agronomic variations among wheat genotypes under conventional and organic systems [23] and on the assessment of essential trace elements in these crops [24]. Al-Ghumaiz et al. [24] evaluated selenium and zinc concentrations in wheat grains and reported genotype-specific differences in response to fertilization methods. However, no data exist on Mg^2+^ dynamics under organic fertilization. Therefore, this study aimed to (1) investigate the impact of organic and conventional fertilization on magnesium content, dry weight, and grain yield in different wheat genotypes (Egyptian genotype (Sids 12), two ICARDA genotypes, two Australian genotypes, Yocora Rojo (YR), and the local genotype (Sama)), (2) assess genotype-specific responses to fertilization types, and (3) identify suitable genotypes for nutritionally adequate wheat production under organic farming conditions in Saudi Arabia.

## 2. Materials and Methods

### 2.1. Site Description and Trial Establishment

Organic (Or) and inorganic (In) fertilizer trials were conducted during the 2020 and 2021 wheat-growing seasons at the Qassim University Agricultural Research and Experimental Station (26°18′28″ N, 43°46′ E). The dominant soil at the site of the experiment was Torriorthents, according to IUSS Working Group WRB [3,25]. Also, the texture of soil was sandy loam, with low levels of soluble salts (EC = 1.5 dSm^−1^) and organic matter (1.3%) and a pH of 8.1. The analysis of the irrigation water revealed an EC of 1.7 dSm^−1^ and a pH of 7.8. Available nutrient values were 28, 12, 35, and 18 mg kg^−1^ for N, P, K, and Mg, respectively, according to [26]. We sowed seven wheat genotypes in a randomized complete block design (RCBD) across three replications, utilizing 3 m^2^ plots (1.5 m × 2 m). These trials comprised seven wheat genotypes (Table 1), with each plot containing 10 rows spaced 25 cm apart. Seeds were sown at 45 kg ha^−1^ on 12 December 2019 and 30 November 2020 for the respective seasons. The trials followed a factorial experiment with randomized complete block design (RCBD) including two factors (fertilization treatments (conventional and organic fertilizations) and wheat genotypes). In the conventional fertilizer trial, urea, diammonium phosphate, and potassium sulfate were applied at rates of 124 kg N ha^−1^, 92 kg P_2_O_2_ ha^−1^, and 57 kg K_2_O ha^−1^. Organic fertilizer, derived from cow manure and applied at 10 t ha^−1^ a month before sowing, was analyzed according to Jones [27], showing an N–P–K–Mg% of 0.5–0.21–0.5–0.02, respectively. Harvests for the first and second growing seasons were performed on 19 April 2020 and 25 April 2021, respectively, by harvesting two rows from each plot.

### 2.2. Measurements

1. Plant height: In the 2021 season, three plants were selected randomly at heading date to measure plant height according to Al-Otayk et al. [28];
2. Shoot dry weight: At the onset of the flowering stage, five plants from each plot were taken randomly in the 2021 season. Shoots were oven-dried at 70 °C for 48 h until a constant weight was achieved to estimate the final shoot dry weight per plant (g) according to Tahoun et al. [29];
3. Grain yield: All plants were harvested from a single square meter for each genotype according to Al-Otayk et al. [28]. The harvested samples were dried and threshed using a thresher machine, and the weight of the grain was determined as a measure of yield in both seasons;
4. Mg content determination: Grain samples of the 2021 season were dried at 70 °C for 48 h, following the protocol by Jones [27]. A 0.2 g sample of the dried tissue was placed in a digestion flask with 5 mL of a 1:3 sulfuric acid/nitric acid mixture. Hydrogen peroxide was also used to facilitate the wet digestion process. Mg content in the digestion extract was measured using a Shimadzu AAS-6000 atomic absorption spectrophotometer, Shimadzu Corporation, Kyoto, Japan.

### 2.3. Statistical Analyses

A factorial experiment within a randomized complete block design (RCBD) was used to assess the effects of fertilization treatments (conventional and organic) and wheat genotypes on various measured parameters. Statistical analyses were performed using JMP Ver. 11 [30]. An analysis of variance (ANOVA) was performed to assess the differences in Mg content in grains, plant dry weight, grain yield, and plant height between organic and conventional fertilizer application across the seven genotypes for both 2020 and 2021 seasons. Duncan’s multiple range tests were used to determine significant differences between treatment means. A linear mixed model was used in which wheat genotypes and fertilization methods factors were regarded as fixed factors. Statistical significance was set at *p* < 0.05. Heatmaps were plotted and analyzed using XLSTAT software version 2019.

## 3. Results

### 3.1. The Main Effect of Fertilizer Application and Wheat Genotypes on Mg Content, Plant Growth, and Grain Yield

We observed significantly higher Mg contents when treating plants with conventional fertilizer (2.12 g kg^−1^) compared with using organic fertilizer (1.54 g kg^−1^) (Table 2). The YR, P3, P5, IC17, and Sids 12 genotypes exhibited high Mg contents, whereas the IC8 and Local genotypes recorded the lowest Mg contents. Conventional fertilization exhibited a higher shoot dry weight per plant (4.6 g) than the organic fertilizer (1.88 g), demonstrating significant effects of both fertilizer application and wheat genotype (Table 2). Sids 12 had the highest shoot dry weight (4.79 g), followed by YR and IC17 (3.39 and 3.31 g, respectively). Significant variances were also observed in grain yield between fertilization treatments and genotypes. The conventional fertilizer yielded significantly higher grain outputs in both 2020 (1.46 t ha^−1^) and 2021 (2.18 t ha^−1^) than the organic fertilizer (0.99 t ha^−1^ in 2020 and 1.81 t ha^−1^ in 2021). Among genotypes, P5 consistently showed the highest grain yield (1.62 t ha^−1^ in 2020 and 2.40 t ha^−1^ in 2021), indicating superior productivity under the tested conditions. Conversely, the Local genotype yielded the least productivity. Sids 12, YR, P3, and IC17 genotypes produced moderate yields. Plant height was significantly influenced by both fertilizer application and genotype, with conventional fertilizer application resulting in taller plants (60.90 cm) than organic fertilization application (54.90 cm). Of the genotypes, IC17 ranked highest in height (62.24 cm), followed by Sids 12 (61.15 cm).

### 3.2. The Effect of the Interaction Between Fertilizer Application and Wheat Genotypes on Mg Content and Grain Yield

**Figure 1:**
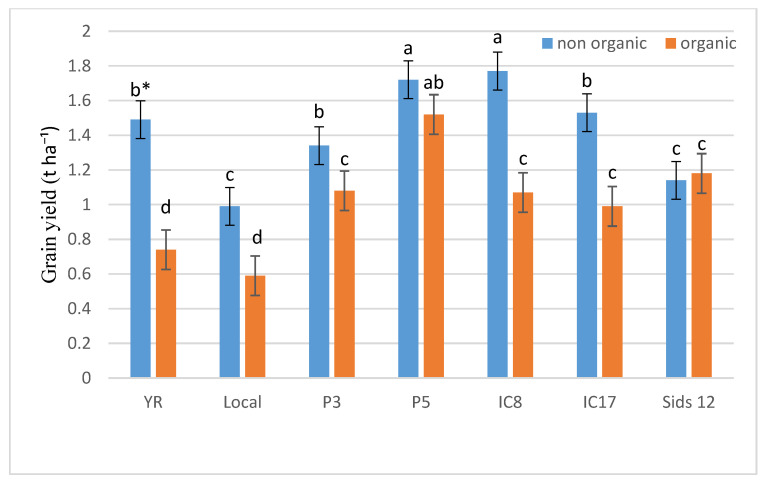
Grain yield (t ha^−1^) of the seven wheat genotypes under conventional and organic fertilization treatments in the 2020 season. * Means separated by same lowercase letters (a, b, c, and d) in the figure were not significant at *p* = 0.05.

**Figure 2:**
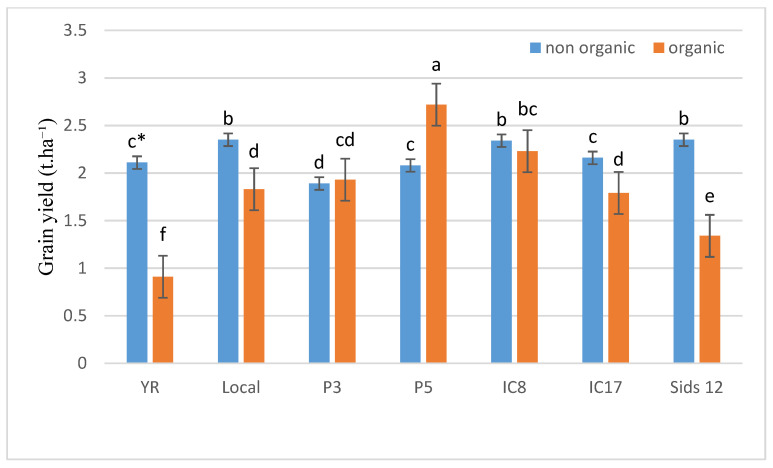
Grain yield (t ha^−1^) of the seven wheat genotypes under conventional and organic fertilization treatments in the 2021 season. * Means separated by same lowercase letters (a, b, c, d, e, and f) in the figure were not significant at *p* = 0.05.

Figure 1 and Figure 2 illustrate the variability in grain yield across genotypes under both inorganic and organic fertilization conditions. Notably, genotypes IC8 and Sids 12 outperformed YR, IC17, and P3 under both conditions. Additionally, the comparative analysis of yields under inorganic versus organic conditions showed that some genotypes, such as YR and IC17, experienced significant yield reductions under organic fertilization, whereas others, such as P5, demonstrated resilience or improved yields. This pattern suggests that certain genotypes might be more suited to organic conditions or benefit more from the specific conditions provided by organic cultivation.

**Figure 3:**
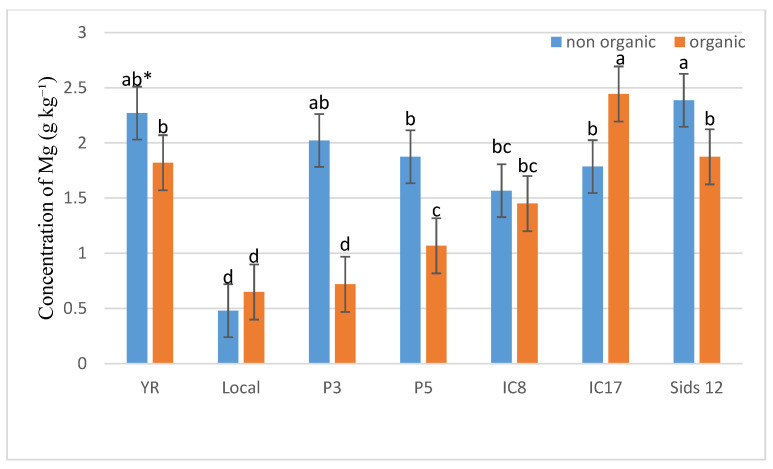
Concentrations of Mg (g kg^−1^) in the grains of the seven wheat genotypes under conventional and organic fertilization treatments in Saudi Arabia. * Means separated by same lowercase letters (a, b, c, and d) in the figure were not significant at *p* = 0.05.

Figure 3 indicates that conventional fertilization enhanced Mg content in wheat grains across various genotypes compared with organic fertilization. For instance, genotype IC17 showed smaller differences between the results of conventional and organic fertilization than genotypes YR or IC8. These findings suggest that genotypes respond differently to various fertilization techniques.

### 3.3. Heatmap Analysis of Overall Data

**Figure 4:**
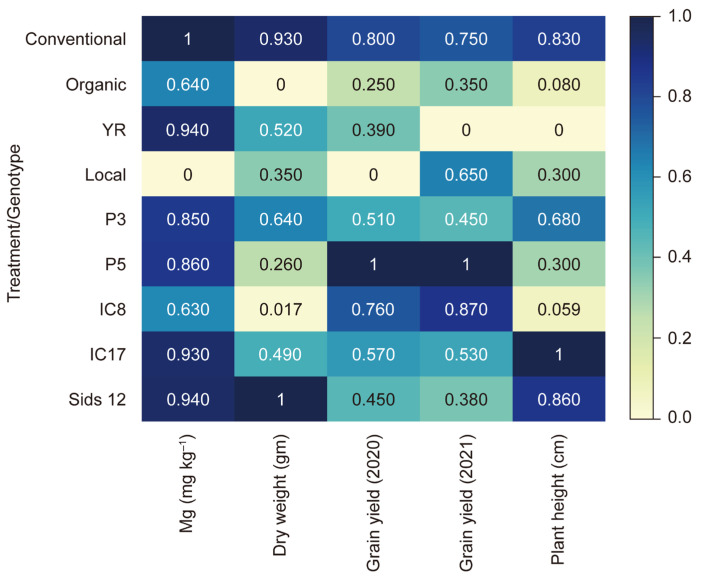
Heatmap of the correlation coefficients between wheat genotypes under organic and conventional inorganic fertilization. Darker colors denote stronger positive correlations, and lighter shades indicate weaker correlations.

We observed correlations between wheat genotypes following organic and conventional fertilization and measured traits such as Mg content, shoot dry weight, grain yield, and plant height (Figure 4). The correlation coefficients varied from 0 to 1, with darker colors denoting stronger positive correlations and lighter shades indicating weaker correlations.

We also observed that the conventional inorganic fertilizer outperformed the organic fertilizer on all measured parameters; however, organic fertilization moderately positively correlated with Mg content (0.64) and grain yield in 2020 (0.35), suggesting a genotype-specific response to organic fertilizer in terms of nutrient uptake and yield. The genotype significantly affected the measured parameters, with genotypes such as P5 and IC17 excelling in grain yield and Mg content, respectively. P5 displayed perfect correlations (1.0) between grain yield and fertilization treatments across 2020 and 2021, illustrating a consistently high performance. These findings emphasize the importance of selecting appropriate agricultural practices and genotypes to maximize wheat production in Saudi Arabia, with conventional farming and genotypes such as P5 and IC17 potentially offering optimal yield and quality.

## 4. Discussion

Organic fertilizers often improve soil health by increasing nutrient availability and enhancing the nutritional quality of crops. Organic fertilization can enhance the content of macronutrients, including Mg, given the improved soil structure and heightened microbial activity [31,32]. However, we observed that Mg content significantly differed between fertilization treatments, with conventional fertilizer producing higher Mg levels than organic fertilizer. This trend is likely attributable to the immediate nutrient availability provided by synthetic fertilizers [33,34]. Organic fertilizers, while sustainable, release nutrients more slowly, possibly explaining the lower Mg concentrations in some genotypes under organic conditions. Magnesium availability in soil is influenced by various cultivation practices, such as soil amendments, crop rotation, and organic matter incorporation, which can enhance Mg release and uptake by plants. The decomposition of organic materials and microbial activity play a crucial role in mineralizing Mg, making it accessible to crops over time. Ryan et al. [33] reported that conventional fertilization, despite enhancing yields, does not necessarily result in higher nutrient concentrations. Therefore, optimizing agronomic practices alongside fertilization strategies is essential for maintaining adequate Mg levels in wheat production.

Lower yields across all genotypes under organic fertilization treatments suggest that inorganic practices involving synthetic fertilizers and pesticides may lead to better productivity. We observed that chemical fertilizer application increased Mg concentration and yield compared with the application of organic treatments. Mg is crucial for chlorophyll formation, and in soils with low fertility, chemical fertilizers such as urea and ammonium phosphate enhance soil fertility [35]. The acidifying effect of urea nitrification is a likely explanation for the increased Mg availability, which could explain the higher Mg content under conventional fertilization. In the research conducted by Tong and Xu [36], it was observed that the addition of urea led to an initial rise in soil pH during the early stages of incubation. This increase was attributed to hydrolysis, which also promoted the growth of ammonia-oxidizing bacteria. As a result, nitrification was enhanced, leading to subsequent soil acidification.

The variation in genotype performance under organic fertilization suggests some genotypes have greater potential for organic cultivation. Differing responses of wheat genotypes to varying fertilization practices have been widely documented. Certain genotypes demonstrate better adaptation to organic fertilization owing to traits such as efficient nutrient uptake and utilization under low-input conditions. While the YR genotype performed well under inorganic fertilization, its substantial yield reduction under organic fertilization suggests it is less suited to such treatments. In contrast, IC17 and Sids 12 exhibited improved stability under organic fertilization, indicating their suitability for organic farming systems. It is important to note that when comparing the effects of different fertilization types with findings from similar studies, variations in results are observed. EL-Guibali [37] reported that applying compost at a rate of 18 t ha^−1^ significantly increased both grain and straw yields, with relative increases of 7.5% and 13.52%, respectively, compared to the control (non-composted) treatment. In contrast, the impact of compost on wheat yield in that study was more pronounced than the effect of organic fertilization in the present study. This difference may be attributed to the compost application rate, which was twice the amount of cow manure used in the current research.

The variation in Mg concentrations among wheat genotypes highlights the role of genetic factors in nutrient uptake and accumulation. Some genotypes display enhanced efficiency in absorbing and storing Mg, particularly under organic conditions. This observation aligns with previous findings showing significant genotype-based variability in nutrient content depending on both genetic composition and fertilization method [38] and with the report that conventional fertilization does not necessarily result in higher nutrient concentrations despite its ability to enhance yields [33]. Genotypes like IC17 and Sids 12 maintained more stable Mg levels across treatments, suggesting better nutrient uptake efficiency or adaptability to organic fertilization. Previous studies have emphasized the importance of selecting genotypes that thrive under organic fertilization, especially given the rising demand for organic products. The variability among genotypes supports earlier findings on genotype-driven nutrient uptake efficiency. Chen et al. [39] reported that certain wheat varieties exhibit superior nutrient extraction from organic sources, likely owing to enhanced root architecture and improved microbial interactions in the rhizosphere. Furthermore, some wheat genotypes perform better in organic systems, with more stable yields and nutrient content under less intensive management [40,41].

Our results showed that wheat genotypes IC17 and Sids 12 offer better performance under organic fertilization conditions in terms of both yield and magnesium content. These results highlight potential trade-offs between yield and magnesium content when transitioning from conventional to organic fertilization systems. In Saudi Arabia, where soil fertility is critical due to the arid conditions, these findings hold particular relevance. Enhancing the nutritional quality of wheat through organic fertilization could improve public health, as Mg plays an essential role in over 300 enzymatic reactions vital for human health [19]. Beyond nutrient content, organic farming practices provide additional benefits, including reduced chemical residues in food, improved soil health, and minimized environmental impact [42].

## 5. Conclusions

In conclusion, our study demonstrates the significant influence of fertilization methods on wheat growth, yield, and magnesium content. Conventional fertilization resulted in higher Mg concentrations, dry weight, and grain yield compared to organic fertilization. However, genotype-specific responses were evident, with IC17 and Sids 12 showing stability in Mg content under organic conditions, while P5 exhibited resilience in grain yield. The study also highlights the role of fertilization in nutrient availability, emphasizing the need for tailored agronomic practices to optimize crop performance. These findings suggest that selecting suitable wheat genotypes can support nutritionally adequate wheat production under organic farming in Saudi Arabia. Further research and breeding programs are essential to enhance nutrient efficiency and yield sustainability in organic systems.

## Figures and Tables

**Table 1:** The seven wheat genotypes used in this study.

Genotype Name	Source
Yocora Rojo (YR) ^†^	USA
Local ^‡^	KSA
P3 (AUS-030851)	Australia
P5 (AUS-030852)	Australia
IC8 (Line-2-ICARDA-1st RDRN0607)	ICARDA ^§^
IC8 (Line-2-ICARDA-1st RDRN0607)	ICARDA ^§^
Sids 12	Egypt

^†^ Yocora Rojo (YR): commercial genotype commonly cultivated in Saudi Arabia. ^‡^ Local genotype (Sama). ^§^ ICARDA: International Center for Agricultural Research in the Dry Areas.

**Table 2:** The effects of fertilization treatment and wheat genotypes on plant height, shoot dry weight, grain yield (across two seasons), and Mg content when grown in Saudi Arabia.

	Plant Height (cm) (2021 Season)	Shoot Dry Weight (g) (2021 Season)	Grain Yield (t ha^−1^) (2020 Season)	Grain Yield (t ha^−1^) (2021 Season)	Mg (Grain) (g kg^−1^) (2021 Season)
Fertilizer application (F)					
Conventional	60.90 a	4.6 a	1.461 a	2.182 a	2.121 a
Organic	54.90 b	1.88 b	0.1995 b	1.818 b	1.54 b
Sig	**	**	**	**	**
Genotype (G)					
YR	54.26 c	3.39 bc	1.117 c	1.507 c	2.01 a
Local	56.64 bc	2.89 cd	0.790 d	2.085 ab	0.51c
P3	59.7 ab	3.74 b	1.213 c	1.907 bc	1.88 a
P5	56.67 bc	2.64 d	1.625 a	2.402 a	1.90 a
IC8	54.73 c	1.93 e	1.423 b	2.285 ab	1.51 b
IC17	62.24 a	3.31 bc	1.265 bc	1.977 ab	2.01 a
Sids 12	61.15 a	4.79 a	1.162 c	1.845 bc	2.09 a
Sig	**	**	**	**	**
F × G	n.s.	**	**	**	**

** Significant at the 0.01 probability level. n.s.: not significant at the 0.05 probability level. As per the Ducan’s multiple range test, groups with the same letter are considered to have the same response, where groups with different letters are considered significantly different.

## Data Availability

All the datasets generated for this study are included in the manuscript.
